# The association between changes in echocardiography and risk of heart failure hospitalizations and death in adults with chronic kidney disease

**DOI:** 10.1038/s41598-023-35440-w

**Published:** 2023-05-31

**Authors:** Jesse K. Fitzpatrick, Rishi V. Parikh, Steven A. Hamilton, Andrew P. Ambrosy, Thida C. Tan, Nisha Bansal, Alan S. Go, for the CRIC Study Investigators, Lawrence J. Appel, Jing Chen, James P. Lash, Robert G. Nelson, Mahboob Rahman, Panduranga S. Rao, Vallabh O. Shah, Raymond R. Townsend, Mark L. Unruh

**Affiliations:** 1 Department of Cardiology, Kaiser Permanente Santa Clara Medical Center, Santa Clara, CA USA; 2 Division of Research, Kaiser Permanente Northern California, 2000 Broadway, Oakland, CA 94612-2304 USA; 3 Department of Epidemiology and Population Health, Stanford University School of Medicine, Palo Alto, CA USA; 4 Department of Cardiology, Kaiser Permanente San Francisco Medical Center, San Francisco, CA USA; 5 Division of Nephrology, Department of Medicine, University of Washington, Seattle, WA USA; 6 Department of Health Systems Science, Kaiser Permanente Bernard J. Tyson School of Medicine, Pasadena, CA USA; 7 Departments of Epidemiology, Biostatistics and Medicine, University of California, San Francisco, San Francisco, CA USA; 8 Department of Medicine, Stanford University, Palo Alto, CA USA; 9 Johns Hopkins University, Baltimore, MD USA; 10 Tulane University, New Orleans, LA USA; 11 University of Illinois at Chicago, Chicago, IL USA; 12 National Institute of Diabetes, Digestive and Kidney Diseases, Bethesda, MD USA; 13 Case Western Reserve University, Cleveland, OH USA; 14 University of Michigan, Ann Arbor, MI USA; 15 University of New Mexico, Albuquerque, NM USA; 16 University of Pennsylvania, Philadelphia, PA USA

**Keywords:** Kidney diseases, Chronic kidney disease

## Abstract

Adults with chronic kidney disease (CKD) are at increased risk for developing heart failure (HF). However, longitudinal cardiac remodeling in CKD has not been well-characterized and its association with HF outcomes remains unknown. We evaluated the association between change in echocardiographic parameters between baseline and year 4 with the subsequent risk of HF hospitalization and death using Cox proportional hazard models in a landmark analysis of a prospective multicenter CKD cohort. Among 2673 participants, mean ± SD age was 61 ± 11 years, with 45% women, and 56% non-white. A total of 472 hospitalizations for HF and 776 deaths occurred during a median (interquartile range) follow-up duration of 8.0 (6.3–9.1) years. Patients hospitalized for HF experienced larger preceding absolute increases in left ventricular (LV) volumes and decreases in LV ejection fraction**.** Adverse changes in LV ejection fraction, LV cavity volume, LV mass index, and LV geometry were independently associated with an increased risk of HF hospitalization and death. Among adults with CKD, deleterious cardiac remodeling occurs over a relatively short timeframe and adverse remodeling is associated with increased risk of HF-related morbidity and mortality.

## Introduction

Chronic kidney disease (CKD) is a growing public health burden affecting > 10% of the worldwide population representing > 800 million individuals^1^. Reduced estimated glomerular filtration rate (eGFR) and proteinuria are independently associated with worse cardiovascular outcomes and a 2-to-threefold higher risk of incident heart failure (HF)^2,3^. Populations with CKD frequently have abnormalities in cardiac structure and function^4–9^, with evidence of adverse cardiac remodeling present even in asymptomatic patients and in those with early kidney disease^7,8,10^. We previously reported that among adults with CKD enrolled in the Chronic Renal Insufficiency Cohort (CRIC) study, baseline echocardiographic findings including enlarged left ventricular (LV) cavity volumes, LV hypertrophy, abnormal LV geometry, reduced LV ejection fraction (LVEF), and/or an enlarged left atrium were associated with an increases risk of HF hospitalization or death^11^.

Cross-sectional studies suggest abnormal cardiac remodeling becomes more severe as eGFR declines^4,6,7,12,13^, but there are limited longitudinal data that describe serial echocardiographic changes in CKD populations over time. The timeframe over which cardiac remodeling occurs in CKD and whether echocardiographic changes are associated with HF-related morbidity and mortality remains unknown. Conversely, it is unclear whether the normalization of pathologic remodeling portends an improved prognosis. A complex interplay between neurohormonal dysregulation and fluid retention inherent to patients with CKD may alter the typical cardiac remodeling observed in HF. As the therapeutic armamentarium of neurohormonal antagonists targeting the cardio-renal axis continues to expand, understanding the complex interrelationship between kidney function and cardiac remodeling will be of paramount importance.

The CRIC study provides a unique opportunity to observe longitudinal echocardiographic changes and clinical outcomes in a large, diverse, and contemporary CKD population with a low rate of prevalent HF. The objectives of this study were (1) to describe the natural history of cardiac remodeling and (2) to assess the independent association between changes in echocardiographic parameters and subsequent risk of HF hospitalization and death in an ambulatory population of adults with CKD.

## Methods

### Study population

The CRIC study was designed to investigate the etiology, prognosis, therapy, health care services utilization, and quality of life among adults with mild-to-moderate CKD. A total of 3939 adult participants with an eGFR between 20 and 70 ml/min per 1.73 m^2^ were initially enrolled between June of 2003 and March of 2007 from seven centers across the United States (Oakland, CA; Ann Arbor, MI; Baltimore, MD; Chicago, IL, Cleveland, OH; New Orleans, LA; and Philadelphia, PA)^14^. Patients were excluded for a history of polycystic kidney disease, use of immunosuppression within the previous 6 months, institutionalization, inability to consent, enrollment in other research studies, pregnancy, New York Heart Association (NYHA) functional class III/IV HF, human immune deficiency virus infection, cirrhosis, myeloma, renal cancer, recent chemotherapy, organ transplant, or chronic dialysis treatment within the last month^14^. Patients without at least 2 echocardiograms (N = 1266, 32%) were further excluded from the present analysis. All participants provided written informed consent, and the study protocol was approved by the Institutional Review Boards of the University of Pennsylvania-Renal Research, John Hopkin’s University ProHealth, University of Maryland, University Hospitals Case Medical Center, MetroHealth Medical Center, The Cleveland Clinic Foundation, University of Michigan Hospital and Health Systems, Wayne State University School of Medicine, University of Illinois at Chicago, Tulane Office of Health Research, Kaiser Permanente Northern California, and the University of California San Francisco. All methods were performed in accordance with the relevant guidelines and regulations.

### Echocardiographic parameters

Baseline and follow-up transthoracic echocardiograms were performed at years 1 and 4 after enrollment using a standardized protocol and transferred to an echocardiography core laboratory at the University of Pennsylvania (Philadelphia, PA) for systematic analysis. The median (interquartile range [IQR]) time between the year 1 and year 4 echocardiogram was 3.0 (2.9–3.1) years. Images were evaluated by a single physician according to contemporaneous guidelines from the American Society of Echocardiography (ASE)^15^. All sonographers performing and physicians interpreting studies in the echocardiography core laboratory were blinded to the participant’s other clinical characteristics.

LV end-diastolic volume (LVEDV) and end-systolic volume (LVESV) were calculated using the biplane method of discs^16^ and indexed to body surface area using the Mosteller formula (LVEDVI and LVESVI, respectively)^17^. LVEF was calculated as (LVEDV-LVESV)/LVEDV × 100 and further stratified into reduced (< 40%), mid-range (40–49%), and preserved LVEF (≥ 50%). LV mass (LVM) was calculated using the area-length method^16^ and indexed to body surface area (LVM index [LMVI]). LV geometry was categorized as normal, concentric remodeling, eccentric hypertrophy, and concentric hypertrophy based on relative wall thickness and LVMI as defined by the ASE^16^. Prevalence of missing echocardiographic variables is provided in the supplement (Supplement, Table 1).

**Table 1:** Characteristics of CRIC study participants stratified by history of HF hospitalization after year 4.

Characteristics	Overall (N = 2673)	HF hospitalization (N = 472)	No HF hospitalization (N = 2201)	*P* value
Demographics				
Mean (SD) age at baseline, years	61.7 (10.6)	64.6 (9.1)	61.1 (10.8)	< 0.001
Women, n (%)	1217 (45.5)	204 (43.2)	1013 (46.0)	0.27
Race/ethnicity, n (%)				< 0.001
Non-Hispanic White	1187 (44.4)	168 (35.6)	1019 (46.3)	
Non-Hispanic Black	1078 (40.3)	239 (50.6)	839 (38.1)	
Hispanic	305 (11.4)	47 (10.0)	258 (11.7)	
Other	103 (3.9)	18 (3.8)	85 (3.9)	
Current smoker, n (%)	251 (9.4)	58 (12.3)	193 (8.8)	< 0.05
Medical history, n (%)				
Heart failure	332 (12.4)	153 (32.4)	179 (8.1)	< 0.001
Prior heart failure hospitalization	219 (8.2)	108 (22.9)	111 (5.0)	< 0.001
Atrial fibrillation or other arrhythmia	560 (21.0)	171 (36.2)	389 (17.7)	< 0.001
Coronary heart disease	743 (27.8)	239 (50.6)	504 (22.9)	< 0.001
Peripheral vascular disease	219 (8.2)	62 (13.1)	157 (7.1)	< 0.001
Stroke	321 (12.0)	87 (18.4)	234 (10.6)	< 0.001
Hypertension	2468 (92.3)	466 (98.7)	2002 (91.0)	< 0.001
Diabetes mellitus	1369 (51.2)	330 (69.9)	1039 (47.2)	< 0.001
Vital signs, mean (SD)				
Body mass index, kg/m^2^	31.9 (7.8)	33.7 (8.7)	31.6 (7.5)	< 0.001
Body surface area, m^2^	2.0 (0.3)	2.1 (0.3)	2.0 (0.3)	< 0.001
Systolic blood pressure, mmHg	127.3 (21.3)	132.4 (23.7)	126.3 (20.7)	< 0.001
Diastolic blood pressure, mmHg	69.0 (12.3)	67.5 (12.8)	69.3 (12.2)	< 0.01
Laboratory values, mean (SD)				
Estimated glomerular filtration rate, mL/min/1.73m^2^	41.7 (18.1)	34.0 (14.8)	43.3 (18.3)	< 0.001
Urine protein-to-creatinine ratio, g/g	0.9 (2.0)	1.3 (2.1)	0.8 (2.0)	< 0.001
High-density lipoprotein, mg/dL	47.0 (15.5)	44.5 (14.7)	47.6 (15.6)	< 0.01
Low-density lipoprotein, mg/dL	98.5 (33.5)	91.1 (33.1)	100.1 (33.4)	< 0.001
Hemoglobin, g/dL	12.6 (1.7)	12.0 (1.7)	12.8 (1.7)	< 0.001
Glycosylated hemoglobin, %	6.5 (1.4)	6.7 (1.4)	6.5 (1.4)	< 0.05
Brain natriuretic peptide, pg/mL (YEAR 1)	75.9 (163.7)	139.3 (279.3)	62.3 (121.5)	< 0.001
Medications				
ACE inhibitors	1139 (42.6)	195 (41.3)	944 (42.9)	0.52
Angiotensin receptor blockers	753 (28.2)	142 (30.1)	611 (27.8)	0.31
Alpha blockers	537 (20.1)	144 (30.5)	393 (17.9)	< 0.001
Beta blockers	1435 (53.7)	346 (73.3)	1089 (49.5)	< 0.001
Calcium channel blockers	1132 (42.4)	239 (50.6)	893 (40.6)	< 0.001
Digoxin	69 (2.6)	27 (5.7)	42 (1.9)	< 0.001
Loop diuretics	951 (35.6)	286 (60.6)	665 (30.2)	< 0.001
Thiazide diuretics	559 (20.9)	79 (16.7)	480 (21.8)	< 0.05
Potassium sparing diuretics	187 (7.0)	51 (10.8)	136 (6.2)	< 0.001
Statins	1693 (63.4)	344 (72.9)	1349 (61.3)	< 0.001
NSAIDs	1504 (56.3)	303 (64.2)	1201 (54.6)	< 0.001
Aspirin	1333 (49.9)	281 (59.5)	1052 (47.8)	< 0.001

### Covariates

Demographic information, medical comorbidities, and medication use was ascertained via self-report questionnaires administered during annual in-person visits and updated during bi-annual telephone visits. Systolic and diastolic blood pressure, heart rate, height, weight, and urine/blood specimens were collected during annual in-person visits using standardized methods. An average of three seated blood pressure measurements was recorded. All laboratory testing was performed in a biomarker core laboratory, and eGFR was calculated using the 2009 Chronic Kidney Disease Epidemiology (CKD-EPI) Collaboration formula^18^. All covariates used in the analysis were obtained at year 4, except for B-type natriuretic peptide, which was only obtained at the time of enrollment.

### Follow-up and outcomes

The primary outcomes of hospitalization for HF and death were collected from the year 4 visit through November 30, 2018. CRIC study personnel screened participants or proxies for hospitalizations and death on a bi-annual basis via telephone interviews. All identified hospitalizations with International Classification of Disease 9th Edition discharge diagnosis codes related to HF (i.e., 398.91, 402.01, 402.11, 402.91, 425.xx, 428.xx, 429.xx, 514.xx, 518.4) were reviewed by two physicians for evidence of documented symptoms, physical exam findings, chest radiographs, echocardiograms, and invasive hemodynamic monitoring if available. A HF hospitalization was considered to have occurred when both reviewers agreed it was “probable” or “definite” based on the modified Framingham clinical criteria^19^. Deaths were also identified through death certificates or obituaries, review of hospital records, and from the Social Security Master File, as available. Participants were censored at the end of follow-up, study withdrawal, or death.

### Statistical analysis

All analyses were conducted using SAS, version 9.4 (Cary, NC, USA) and R, version 4.0.2 (https://www.r-project.org/). Baseline characteristics were collected at year 4 (i.e., second echocardiogram) for this landmark analysis and were stratified by the presence or absence of HF hospitalization. Categorical variables are presented as frequencies with percentages and continuous variables as means with standard deviations. We describe the changes from year 1 to year 4 as both absolute and relative differences for continuous variables, and category changes for categorical variables. Differences between baseline characteristics and changes in echocardiographic parameters were compared using analysis of variance or Kruskal–Wallis tests for continuous variables and Chi-square tests for categorical variables.

We first used Kaplan–Meier survival curves to assess differences in hospitalization for HF and all-cause death among participants stratified by the level and direction of 3-year changes in LVEF, LVMI, LVESVI index, and LVEDVI. Cut points for Kaplan–Meier curves were defined using 10% of the population mean value at year 1; for example, if the mean LVEF at year 1 was 50%, we categorized individuals as having “No Change” if the difference in LVEF from year 1 to 4 was between -5% and 5%, an “Increase” if the difference in LVEF was > 5%, or a “Decrease” if the difference in LVEF was < − 5%. Next, we evaluated the independent associations between 3-year absolute changes in echocardiographic measurements and the outcomes of interest using multivariable Cox proportional hazard models controlling for potential confounders at year 4, including demographics (age, sex, race/ethnicity), medical history (presence of HF, HF hospitalizations before the year 4 study visit, atrial fibrillation, acute myocardial infarction, coronary revascularization, peripheral vascular disease, stroke, tobacco use, alcohol use), vital signs (body mass index, systolic blood pressure), and laboratory values (eGFR by CKD-EPI equation, low-density lipoprotein cholesterol, hemoglobin, and glycosylated hemoglobin), and echocardiographic parameter value at baseline (i.e., year 1), and medical therapy for HF (beta blockers, loop diuretics, and potassium sparing diuretics). We included a random effect for CRIC clinical center to account for within-center correlation and used a multiple imputation approach across 50 imputed datasets to account for missing data in echocardiographic and laboratory variables. Finally, an interaction analysis was performed between change in echocardiographic parameters and markers of kidney function (eGFR and urine protein-to-creatinine ratio) for the outcomes of HF hospitalization and death.

## Results

Among 2,673 participants with serial echocardiograms, mean age was 61.7 ± 10.6 years, 45% were women, and 56% self-identified as non-white (Table 1). There was a high burden of cardiovascular and non-cardiovascular disease, with 12.4% of participants diagnosed with HF. Participants hospitalized for HF were more likely to be older, non-white, and had a higher burden of medical comorbidities. The average eGFR was lower among those hospitalized for HF, while glycosylated hemoglobin and brain natriuretic peptide were higher (Table 1).

**Figure 1:**
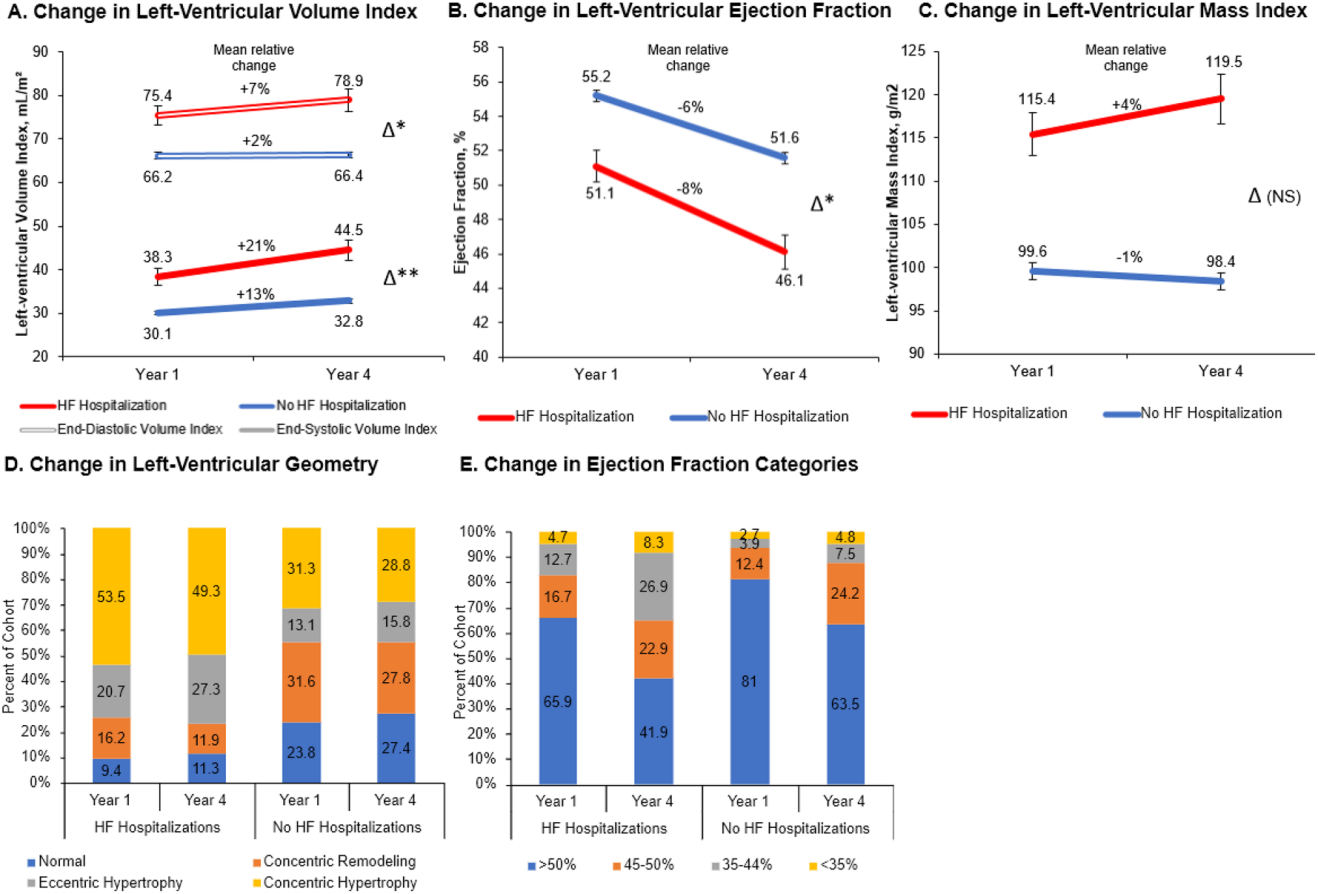
Absolute and relative (% change from baseline) change in (**A**) left ventricular volume indexes, (**B**) left ventricular ejection fraction, (**C**) left ventricular mass index, (**D**) categorical left ventricular geometry, and (**E**) categorial left ventricular ejection fraction between year 1 and year 4 echocardiogram, stratified by presence or absence of heart failure hospitalization after year 4. HF, heart failure.

**Figure 2:**
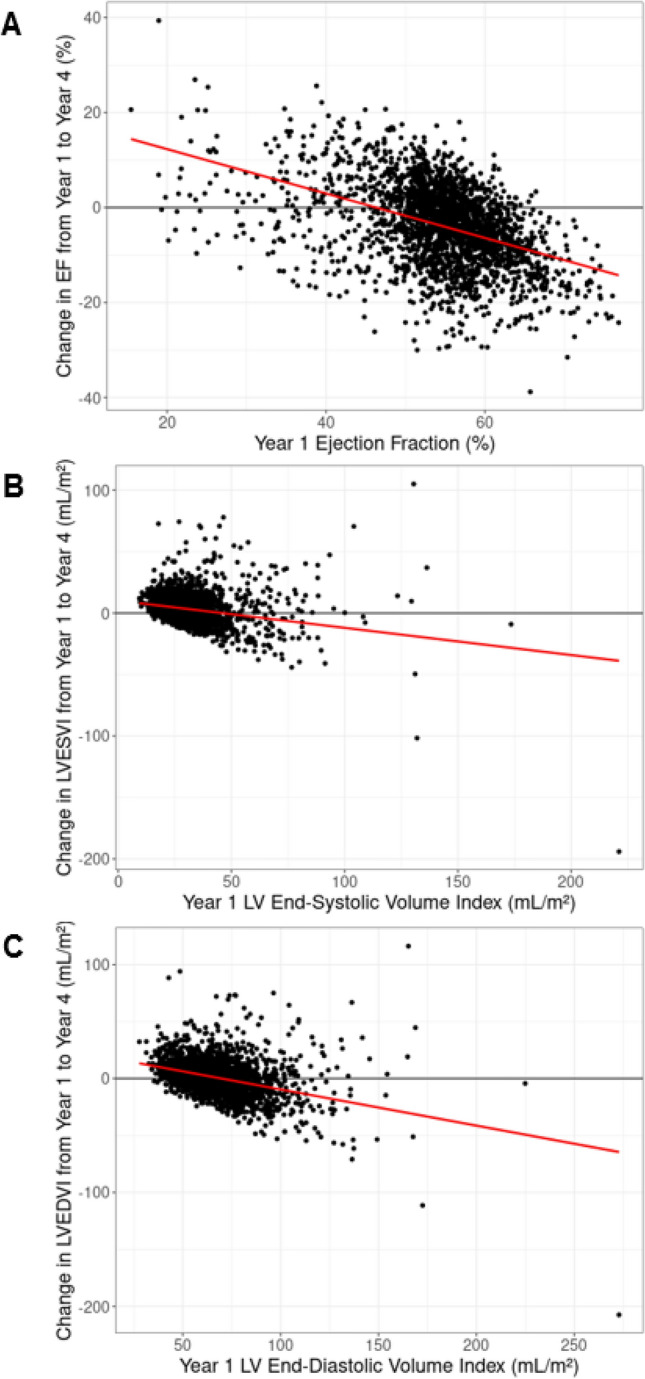
Scatter plot of baseline (**A**) ejection fraction, (**B**) end systolic volume index, and (**C**) end-diastolic volume index plotted against the change in the same echocardiographic measure between year 1 and year 4. EF, ejection fraction; LV, left ventricular; LVESVI, left ventricular end-systolic volume index; LVEDVI, left ventricular end-diastolic volume index.

Year 1 and year 4 echocardiographic parameters, stratified by subsequent HF hospitalization status, are presented in Table 2. Patients hospitalized for HF after year 4 had more severe preceding abnormalities in cardiac structure and function. Between year 1 and year 4, average LV volumes increased, while mean LVEF declined across the cohort, but the magnitude of change was larger in those with a subsequent HF hospitalization (Table 3, Fig. 1). Mean LVMI increased in patients subsequently hospitalized for HF and declined in those without a hospitalization for HF, although the differences were not statistically significant. Eccentric hypertrophy became more common over the study period, especially among those who experienced a subsequent HF hospitalization. Participants with more severe baseline cardiac abnormalities (i.e., lower LVEF and higher LVESV/LVEDV) experienced greater improvements in LVEF and decreases in LV volumes between year 1 and year 4 (Fig. 2).

**Table 2:** Echocardiographic measurements stratified by history of HF hospitalization after year 4.

Characteristics	Overall (N = 2673)	HF hospitalization (N = 472)	No HF hospitalization (N = 2201)	*P* value
Year 1 echo measurements				
Left ventricular ejection fraction, %, categorical				< 0.001
≥ 50%	2094 (78.3)	311 (65.9)	1783 (81.0)	
40–49%	352 (13.2)	79 (16.7)	273 (12.4)	
< 40%	146 (5.5)	60 (12.7)	86 (3.9)	
Left ventricular ejection fraction, %	54.5 (8.1)	51.1 (10.2)	55.2 (7.4)	< 0.001
Left ventricular mass indexed to BSA, g/m^2^	102.3 (24.4)	115.4 (27.3)	99.6 (22.9)	< 0.001
Left ventricular end-systolic volume indexed to BSA, mL/m^2^	31.5 (13.7)	38.3 (20.8)	30.1 (11.1)	< 0.001
Left ventricular end-diastolic volume indexed to BSA, mL/m^2^)	67.8 (17.8)	75.4 (24.0)	66.2 (15.8)	< 0.001
Left ventricular geometry				< 0.001
Normal	474 (17.7)	36 (7.6)	438 (19.9)	
Concentric remodeling	645 (24.1)	62 (13.1)	583 (26.5)	
Eccentric hypertrophy	321 (12.0)	79 (16.7)	242 (11.0)	
Concentric hypertrophy	781 (29.2)	204 (43.2)	577 (26.2)	
Left atrial four chamber area, cm^2^	23.1 (5.5)	25.6 (6.0)	22.6 (5.2)	< 0.001
Year 4 echo measurements				
Left ventricular ejection fraction, categorical				< 0.001
> 50%	1595 (59.7)	198 (41.9)	1397 (63.5)	
40–49%	641 (24.0)	108 (22.9)	533 (24.2)	
< 40%	292 (10.9)	127 (26.9)	165 (7.5)	
Left ventricular ejection fraction, %	50.6 (8.8)	46.1 (11.3)	51.6 (7.8)	< 0.001
Left ventricular mass indexed to BSA, g/m^2^	102.0 (26.0)	119.5 (31.4)	98.4 (23.3)	< 0.001
Left ventricular end-systolic volume indexed to BSA (mL/m^2^)	34.8 (16.3)	44.5 (25.6)	32.8 (12.7)	< 0.001
Left ventricular end-diastolic volume indexed to BSA (mL/m^2^)	68.5 (19.4)	78.9 (27.9)	66.4 (16.3)	< 0.001
Left ventricular geometry				< 0.001
Normal	541 (20.2)	42 (8.9)	499 (22.7)	
Concentric remodeling	550 (20.6)	44 (9.3)	506 (23.0)	
Eccentric hypertrophy	390 (14.6)	101 (21.4)	289 (13.1)	
Concentric hypertrophy	707 (26.4)	182 (38.6)	525 (23.9)	
Left atrial four chamber area, cm^2^	24.1 (5.8)	26.9 (6.8)	23.5 (5.4)	< 0.001

**Table 3:** Changes in echocardiographic parameters between year 1 and 4 stratified by history of HF hospitalization after year 4.

Echocardiogram measurement	Absolute differences from Y1 to Y4			Relative differences (% change Y1 to Y4)		
	HF hospitalizations (N = 472)	No HF hospitalizations (N = 2201)	*P* value	HF hospitalizations (N = 472)	No HF hospitalizations (N = 2201)	*P* value
Left ventricular ejection fraction, category change n (%)			< 0.001	N/A	N/A	N/A
No change	237 (50.2)	1369 (62.2)		N/A	N/A	N/A
Increase in ejection fraction (improvement)	32 (6.8)	162 (7.4)		N/A	N/A	N/A
Decrease in ejection fraction (worsening)	150 (31.8)	523 (23.8)		N/A	N/A	N/A
Left ventricular ejection fraction, %	− 4.73 (9.87)	− 3.63 (8.13)	< 0.05	− 7.5 (24.3)	− 5.6 (15.5)	0.13
Left ventricular mass indexed to BSA	1.88 (26.90)	− 0.79 (19.61)	0.08	3.5 (23.3)	0.9 (19.4)	0.06
Left ventricular end-systolic volume (ml) indexed to BSA	5.81 (20.33)	2.64 (10.11)	< 0.01	21.3 (48.5)	13.3 (35.0)	** < 0.01**
Left ventricular end-diastolic volume (ml) indexed to BSA	3.13 (23.55)	0.10 (13.95)	< 0.05	6.9 (29.2)	2.2 (21.3)	** < 0.01**
Left ventricular geometry (category change)			< 0.001	N/A	N/A	N/A
No change	174 (36.9)	812 (36.9)		N/A	N/A	N/A
Normal to abnormal	14 (3.0)	184 (8.4)		N/A	N/A	N/A
Abnormal to normal	25 (5.3)	237 (10.8)		N/A	N/A	N/A
Abnormal to abnormal	108 (22.9)	369 (16.8)		N/A	N/A	N/A
Left atrial four chamber area	1.25 (6.04)	0.94 (5.04)	0.31	7.2 (24.8)	6.7 (23.6)	0.66

Absolute differences represented as means (SD) for continuous variables and change in category for categorical variables. Left ventricular categories are > 50%, 40–49%, and < 40%. Abnormal left ventricular geometry included left ventricular remodeling, eccentric hypertrophy, and eccentric hypertrophy.

**Figure 3:**
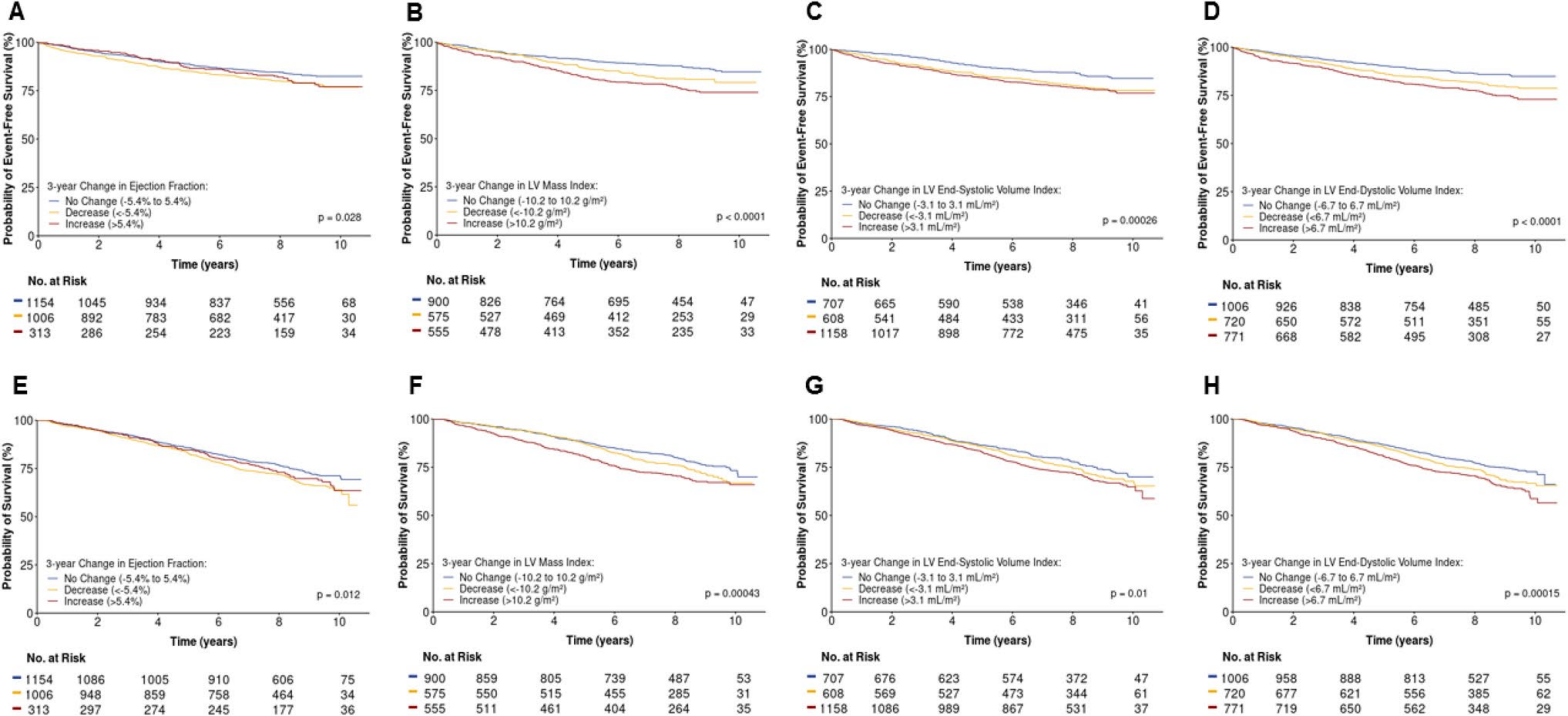
Kaplan–Meier event-free survival from heart failure hospitalization (**A**–**D**) and death (**E**–**H**) stratified by absolute change in (**A**/**E**) left ventricular ejection fraction, (**B**/**F**) left ventricular mass index, (**C**/**G**) left ventricular end-systolic volume index, and (**D**/**H**) left ventricular end-diastolic volume index. Event-free survival estimates stratified by no change, increase, or decrease in the echocardiographic parameter defined as an absolute change corresponding to > 10% of the cohort average.

During a median (IQR) of follow-up of 8.0 (6.3–9.1) years, 18% of participants were hospitalized for HF (2.6 per 100 person-years, 95% confidence interval [CI] 2.4, 2.8) and 29% died (4.0 per 100 person-years, 95% CI 3.7, 4.3). Kaplan–Meier curves of outcomes stratified by change (i.e., increase, decrease, no change) in LVEF, LVMI, LVESVI, and LVEDVI are shown in Fig. 3. A decline in LVEF, or an increase in LVMI, LVESVI, and LVEDVI was associated with a lower crude probability of event-free survival (i.e., HF hospitalization or death). However, the opposite (i.e., favorable cardiac remodeling) did not reliably predict better outcomes, compared to the reference group (i.e., no significant change).

After adjustment for clinical confounders, baseline echocardiographic parameters, and medical therapy for HF, adverse changes in echocardiographic measures were generally associated with an increased risk of HF hospitalizations and, to a lesser extent, death (Table 4). Decreases in LVEF and increase in LVMI, LVESVI, LVEDVI, and left atrial area were independently associated with higher risks of both outcomes. Normalization of LV geometry was associated with a lower risk of both HF hospitalization and death, while an improvement in LVEF category was associated with a lower risk of HF hospitalization. There was no interaction between the change in echocardiographic parameters and baseline eGFR and urine protein-to-creatinine ratio for the outcomes of HF hospitalization and death (Supplement, Table 2).

**Table 4:** Associations between changes in echocardiogram measurements and outcomes in multivariable* Cox proportional hazards models.

Change in echocardiogram variable (per 1 SD increase in the delta from Y1 to Y4)	Hazard ratio (95% CI)					
	Heart failure hospitalization			Death		
	Adjusting for clinical confounders only	Clinical confounders + Y1 echo value	Clinical confounders + Y1 echo value + medications	Adjusting for clinical confounders only	Clinical confounders + Y1 echo value	Clinical confounders + Y1 echo value + medications
Left ventricular ejection fraction (EF), categorical						
No change in category	Ref	Ref		Ref	Ref	Ref
Improvement in EF	1.09 (0.75–1.58)	0.55 (0.38–0.80)	0.80 (0.54–1.17)	1.30 (1.00–1.68)	0.77 (0.57–1.02)	0.94 (0.70–1.28)
Worsening EF	1.53 (1.25–1.87)	1.74 (1.41–2.14)	1.61 (1.30–2.00)	1.24 (1.06–1.46)	1.34 (1.14–1.58)	1.19 (1.00–1.42)
Left ventricular EF (per 1 SD decrease in delta)	1.12 (1.02–1.23)	1.47 (1.33–1.61)	1.28 (1.16–1.43)	1.06 (0.99–1.15)	1.25 (1.16–1.35)	1.13 (1.04–1.22)
Left ventricular mass indexed to BSA	1.15 (1.03–1.28)	1.46 (1.33–1.59)	1.32 (1.20–1.46)	1.07 (0.99–1.15)	1.26 (1.18–1.35)	1.12 (1.04–1.21)
Left ventricular end-systolic volume indexed to BSA	1.31 (1.20–1.44)	1.22 (1.16–1.29)	1.17 (1.10–1.25)	1.14 (1.06–1.23)	1.15 (1.09–1.21)	1.08 (1.02–1.15)
Left ventricular end-diastolic volume indexed to BSA	1.23 (1.11–1.36)	1.27 (1.19–1.35)	1.18 (1.09–1.26)	1.10 (1.02–1.18)	1.19 (1.12–1.26)	1.07 (1.00–1.15)
Left ventricular geometry						
No change	Ref	Ref		Ref	Ref	Ref
Normal to abnormal	0.37 (0.23–0.60)	0.81 (0.44–1.50)	0.79 (0.42–1.49)	0.70 (0.58–1.01)	1.23 (0.80–1.87)	1.33 (0.85–2.08)
Abnormal to normal	0.55 (0.37–0.80)	0.62 (0.41–0.94)	0.90 (0.59–1.37)	0.76 (0.58–1.01)	0.73 (0.54–0.98)	0.95 (0.70–1.28)
Abnormal to abnormal	1.45 (1.17–1.79)	1.10 (0.87–1.39)	1.07 (0.84–1.36)	1.31 (1.10–1.55)	1.08 (0.89–1.31)	1.07 (0.88–1.31)
Left atrial four chamber area	1.07 (0.98–1.18)	1.35 (1.24–1.47)	1.19 (1.08–1.30)	1.05 (0.97–1.13)	1.21 (1.13–1.30)	1.10 (1.01–1.19)

*Each model adjusted for: age, sex, race/ethnicity, tobacco use, alcohol use, body mass index, systolic blood pressure, prior atrial fibrillation, acute myocardial infarction, coronary artery revascularization, peripheral vascular disease, stroke, heart failure, estimated glomerular filtration rate, urine protein-to-creatinine ratio, LDL cholesterol, hemoglobin, glycosylated hemoglobin, HF hospitalization prior to Year 1, HF hospitalization between Year 1 and Year 4. Medications include beta blockers, loop diuretics, and potassium-sparing diuretics.

## Discussion

In this prospective study of 2673 adults with mild-to-moderate CKD at baseline, we observed significant cardiac remodeling over a 3-year time period with reductions in LVEF and increases in LV volumes, particularly LVESV. Participants with more severe baseline LV cavity dilation and systolic dysfunction experienced the greatest improvements (i.e., normalization) in echocardiographic parameters during the 3-year period. Participants subsequently hospitalized for HF experienced more adverse cardiac remodeling (i.e., reduction in LVEF and increase in LV volume) over the preceding years. There was a trend toward increased LVMI in subjects hospitalized for HF and they were more likely to develop eccentric LV hypertrophy than those not hospitalized for HF. In fully adjusted models that accounted for baseline clinical characteristics and echocardiographic parameters, adverse changes in LV volumes, LVEF, and LVMI were all independently associated with increased risk of HF hospitalizations and death. These findings suggest serial echocardiograms over a timeframe of several years can detect cardiac remodeling in individuals with CKD, and that even modest changes in cardiac structure and function can be prognostically meaningful for outcomes of HF-related morbidity and mortality.

To our knowledge, this is the first study to prospectively report longitudinal echocardiographic changes in a large contemporary cohort with CKD. Serial echocardiographic measurements among the subset of participants in the CRIC study who progressed to ESKD has been previously reported^20,21^. Compared to our larger cohort, the 417 patients who progressed to ESKD had modest but significant declines in LVMI and experienced no change in LVEDVI, but other echocardiographic parameters were consistent with our findings. In a smaller study of 98 patients with stage 5 CKD, LVMI and LV geometry did not change appreciably over a 2-year study period^22^. The mean baseline eGFR of participants who developed ESKD during the CRIC study was predictably lower than the overall cohort (24.6 vs. 41.7 ml/min/1.73 m^2^). The discovery of distinct patterns of cardiac remodeling at different eGFR ranges within the same cohort suggests that the relationship between eGFR and cardiac remodeling may change as CKD progresses. Alternatively, other clinical circumstances associated with advanced CKD may influence cardiac remodeling in a manner that opposes the effect of CKD (i.e., effect modification). For example, an elevated glycosylated hemoglobin is independently associated with abnormal cardiac remodeling^23^, but in advanced CKD, reduced insulin clearance contributes to lower glycosylated hemoglobin levels^24^. Data from cross-sectional studies, which are more vulnerable to this type of effect modification, are heterogenous but have generally shown that markers of deleterious cardiac remodeling worsen as eGFR declines^4,6,12^. Serial echocardiograms across a longer time horizon could detect non-linear changes in cardiac structure and function and provide a more comprehensive understanding of cardiac remodeling across a wide spectrum of eGFR.

In clinical practice, reversal of abnormal echocardiographic findings is typically interpreted as a favorable prognostic finding. Given this assumption, some of our findings may at first seem counterintuitive. In our Kaplan–Meier analysis**,** participants with seemingly favorable cardiac remodeling (i.e., increases in LVEF and decreases in LVESVI or LVEDVI) consistently experienced higher crude rates of HF hospitalization and death than subjects with no change in their echocardiogram. However, after adjustment for baseline echocardiographic parameters, an improvement in LVEF category and normalization of LV geometry had strong favorable associations with subsequent HF hospitalization and death. Review of participant-level data reveals that subjects with lower baseline LVEF and larger LV volumes experienced more substantial improvement in echocardiographic measures. Measurement error may partially explain this finding, but a physiologic mechanism is also possible. A potential explanation is that adults with more abnormal baseline echocardiography were aggressively treated with guideline directed medical therapy for HF which had beneficial effects on cardiac remodeling. Regardless of mechanism, the ultimate consequence of this trend was that subjects with more favorable echocardiographic changes also had more abnormal echocardiograms and therefore appeared to have worse outcomes in analysis that did not account for baseline echocardiography. Our findings highlight the critical importance of interpreting changes in echocardiography within the clinical context of baseline values.

Our study had several limitations. The CRIC study excluded subjects with NYHA functional class III/IV HF, which likely reduced participation among adults with prevalent HF and reduced systolic function and lowered the event rate for HF hospitalizations and potentially death. However, this decision allowed us to describe the natural history of echocardiographic findings in the setting of mild-to-moderate CKD patients with a low rate of prevalent HF which is unique. The protocol required echocardiograms at only two time points separated by a median of 3 years, potentially limiting our ability to detect non-linear associations between CKD progression and cardiac remodeling. Low rates of moderate to severe valvular heart disease at baseline precluded analyzing these findings. The approximately 20% of CRIC participants who did not have a year 4 echocardiogram were sicker than our analytical cohort (Supplement, Table 3) and approximately 40% died prior to year 4. Therefore, our results may not be fully representative of critically ill patients or those near the end of life. Contemporary measures of diastolic function such as tissue Doppler imaging and estimates of pulmonary artery systolic pressure were not collected and limited our ability to analyze diastology. However, we did include left atrial area which is an important marker of chronically elevated left-sided filling pressures. Finally, adjudication of HF hospitalizations did not require a change in HF-related therapy, which may have reduced the diagnostic specificity of the operational definition for this endpoint.

In conclusion, we found that deleterious cardiac remodeling occurs over a relatively short time horizon in the setting of mild-to-moderate CKD and that adults subsequently hospitalized for HF experienced larger preceding absolute decreases in LV systolic function and increases in LV volumes. Furthermore, adverse changes in LVEF, LV volumes, LVMI, and LV geometry were all independently associated with increased risks of subsequent HF hospitalization and death. Effective blood pressure control and guideline directed medical therapy for HF, when indicated, may prevent deleterious cardiac remodeling and improve outcomes in CKD populations. Echocardiography can help identify patients at high risk for HF-related morbidity, and the potential benefit of routine echocardiography in CKD populations should be investigated in prospective trials. Future studies should also explore potential non-linear cardiac remodeling in CKD and further investigate the prognostic significance of (pseudo)normalization of echocardiographic parameters.

## Supplementary Information

Supplementary Information.

## Data Availability

The datasets generated during and/or analyzed during the current study are available from the corresponding author on reasonable request.
