# Supplementary material for: The association between changes in echocardiography and risk of heart failure hospitalizations and death in adults with chronic kidney disease
Source: Sci Rep. 2023 May 31;13:8863. doi: 10.1038/s41598-023-35440-w (PMC10232430; doi:10.1038/s41598-023-35440-w)
Supplement: Supplementary file 1 — Supplementary Information. [file 41598_2023_35440_MOESM1_ESM.docx]

**Supplement**

**Supplemental Table 1. Prevalence of missingness of echocardiographic variables among participants who did or did not experience a heart failure hospitalization.**

|  | Overall  (N=2673) | HF Hospitalization  (N=472) | No HF Hospitalization  (N=2201) |
| --- | --- | --- | --- |
| **Year 1 Echocardiogram Missing, n (%)** |  |  |  |
| Left ventricular ejection fraction, %, categorical | 81 (3.0) | 22 (4.7) | 59 (2.7) |
| Left ventricular ejection fraction, % | 81 (3.0) | 22 (4.7) | 59 (2.7) |
| Left ventricular mass indexed to BSA, g/m^2^ | 358 (13.4) | 75 (15.9) | 283 (12.9) |
| Left ventricular end-systolic volume indexed to BSA (mL/m^2^) | 81 (3.0) | 22 (4.7) | 59 (2.7) |
| Left ventricular end-diastolic volume indexed to BSA (mL/m^2^) | 77 (2.9) | 20 (4.2) | 57 (2.6) |
| Left ventricular geometry | 452 (16.9) | 91 (19.3) | 361 (16.4) |
| Left atrial four chamber area | 42 (1.6) | 9 (1.9) | 33 (1.5) |
| **Year 4 Echocardiogram Missing, n (%)** |  |  |  |
| Left ventricular ejection fraction, %, categorical | 145 (5.4) | 39 (8.3) | 106 (4.8) |
| Left ventricular ejection fraction, % | 145 (5.4) | 39 (8.3) | 106 (4.8) |
| Left ventricular mass indexed to BSA, g/m^2^ | 426 (15.9) | 93 (19.7) | 333 (15.1) |
| Left ventricular end-systolic volume indexed to BSA (mL/m^2^) | 146 (5.5) | 39 (8.3) | 107 (4.9) |
| Left ventricular end-diastolic volume indexed to BSA (mL/m^2^) | 122 (4.6) | 35 (7.4) | 87 (4.0) |
| Left ventricular geometry | 485 (18.1) | 103 (21.8) | 382 (17.4) |
| Left atrial four chamber area | 60 (2.2) | 17 (3.6) | 43 (2.0) |

**Table 2. Interaction analyses between change in echocardiographic measures between year 1 and 4 and markers of kidney function for outcomes of heart failure hospitalization and death in adults with chronic kidney disease.**

| **Change in echocardiogram variable (per 1 SD increase in the delta from Y1 to Y4)** | **P-value for interaction term** | | | |
| --- | --- | --- | --- | --- |
|  | **Heart Failure Hospitalization** | | **Death** | |
|  | Interaction with estimated glomerular filtration rate | Interaction with urinary protein-to-creatine ratio | Interaction with estimated glomerular filtration rate | Interaction with urinary protein-to-creatine ratio |
| Left ventricular ejection fraction | 0.84 | 0.73 | 0.81 | 0.28 |
| Left ventricular mass indexed to BSA | 0.77 | 0.60 | 0.85 | 0.41 |
| Left ventricular end-systolic volume indexed to BSA | 0.07 | 0.23 | 0.81 | 0.62 |
| Left ventricular end-diastolic volume indexed to BSA | 0.05 | 0.10 | 0.97 | 0.78 |
| Left ventricular geometry | 0.53 | 0.49 | 0.64 | 0.48 |
| Left atrial four chamber area | 0.23 | 0.24 | 0.10 | 0.11 |
| *Each model adjusted for: age, sex, race/ethnicity, tobacco use, alcohol use, body mass index, systolic blood pressure, prior atrial fibrillation, acute myocardial infarction, coronary artery revascularization, peripheral vascular disease, stroke, heart failure, estimated glomerular filtration rate, urine protein-to-creatinine ratio, LDL cholesterol, hemoglobin, glycosylated hemoglobin, heart failure hospitalization prior to Year 1, heart failure hospitalization between Year 1 and Year 4. | | | | |

| **Table 3. Baseline characteristics at year 1 of participants with and without a year 4 echocardiogram.** | | | | | |
| --- | --- | --- | --- | --- | --- |
| **Characteristics** | **Year 4 Echocardiogram**  (N=2673) | **No Year 4 Echocardiogram**  (N=832) | **P-Value** | **Standardized Difference** |  |
| **Demographic characteristics** |  |  |  |  |  |
| Age at baseline |  |  |  |  |  |
| Mean (SD) | 58.7 (10.7) | 59.4 (11.2) | 0.1 | 0.06 |  |
| Median (interquartile range) | 60.0 (53.0-66.0) | 61.5 (53.0-68.0) | **<0.05** |  |  |
| Range | 22.0-76.0 | 22.0-76.0 |  |  |  |
| Sex |  |  | 0.64 | 0.01 |  |
| Male | 1456 (54.5) | 461 (55.4) |  |  |  |
| Female | 1217 (45.5) | 371 (44.6) |  |  |  |
| Race/ethnicity |  |  | **<0.001** | 0.09 |  |
| Non-Hispanic White | 1187 (44.4) | 310 (37.3) |  |  |  |
| Non-Hispanic Black | 1078 (40.3) | 340 (40.9) |  |  |  |
| Hispanic | 305 (11.4) | 148 (17.8) |  |  |  |
| Other | 103 (3.9) | 34 (4.1) |  |  |  |
| **Current smoker** | 291 (10.9) | 130 (15.6) | **<0.001** | 0.06 |  |
| **Alcohol use** | 1608 (60.2) | 434 (52.2) | **<0.001** | 0.07 |  |
| **Medical History, n (%)** |  |  |  |  |  |
| Atrial fibrillation or heart arrhythmia | 469 (17.5) | 169 (20.3) | 0.07 | 0.03 |  |
| MI/prior revascularization | 587 (22.0) | 225 (27.0) | **<0.01** | 0.05 |  |
| Peripheral vascular disease | 165 (6.2) | 92 (11.1) | **<0.001** | 0.08 |  |
| Stroke | 275 (10.3) | 93 (11.2) | 0.46 | 0.01 |  |
| Congestive heart failure | 240 (9.0) | 126 (15.1) | **<0.001** | 0.09 |  |
| Cardiovascular disease | 900 (33.7) | 341 (41.0) | **<0.001** | 0.07 |  |
| Hypertension | 2349 (87.9) | 756 (90.9) | **<0.05** | 0.04 |  |
| Diabetes mellitus | 1266 (47.4) | 482 (57.9) | **<0.001** | 0.09 |  |
| Anemia | 1112 (41.6) | 418 (50.3) | **<0.001** | 0.07 |  |
| Chronic Obstructive Pulmonary Disease | 114 (4.3) | 52 (6.3) | **<0.05** | 0.04 |  |
| **Vital signs** |  |  |  |  |  |
| Body mass index, kg/m^2^ |  |  |  |  |  |
| Mean (SD) | 32.0 (7.6) | 32.1 (7.9) | 0.78 | 0.01 |  |
| Median (interquartile range) | 30.8 (26.7-35.9) | 30.8 (26.6-36.5) | 0.95 |  |  |
| Range | 15.4-85.3 | 14.3-79.4 |  |  |  |
| Missing, n (%) | 1 (0.0) | 2 (0.2) |  |  |  |
| Calculated body surface area, m^2^ |  |  |  |  |  |
| Mean (SD) | 2.1 (0.3) | 2.0 (0.3) | 0.63 | 0.02 |  |
| Median (interquartile range) | 2.0 (1.9-2.2) | 2.0 (1.8-2.2) | 0.61 |  |  |
| Range | 1.2-3.3 | 1.3-3.1 |  |  |  |
| Missing, n (%) | 1 (0.0) | 2 (0.2) |  |  |  |
| Systolic blood pressure, mmHg |  |  |  |  |  |
| Mean (SD) | 126.4 (21.0) | 130.3 (23.5) | **<0.001** | 0.18 |  |
| Median (interquartile range) | 123.3 (111.3-138.0) | 126.7 (114.7-144.0) | **<0.001** |  |  |
| Range | 68.7-238.0 | 74.7-237.3 |  |  |  |
| Missing, n (%) | 0 (0.0) | 0 (0.0) |  |  |  |
| Diastolic blood pressure, mmHg |  |  |  |  |  |
| Mean (SD) | 70.3 (12.7) | 69.5 (12.9) | 0.13 | 0.06 |  |
| Median (interquartile range) | 69.3 (61.3-78.7) | 69.3 (60.0-78.7) | 0.2 |  |  |
| Range | 30.0-123.3 | 38.0-128.0 |  |  |  |
| Missing, n (%) | 0 (0.0) | 0 (0.0) |  |  |  |
| Seated heart rate, beats/min |  |  |  |  |  |
| Mean (SD) | 67.9 (11.3) | 68.6 (11.5) | 0.12 | 0.06 |  |
| Median (interquartile range) | 66.0 (60.0-76.0) | 66.0 (60.0-76.0) | 0.19 |  |  |
| Range | 31.0-112.0 | 38.0-110.0 |  |  |  |
| Missing, n (%) | 0 (0.0) | 0 (0.0) |  |  |  |
| **Laboratory values** |  |  |  |  |  |
| Estimated glomerular filtration rate, mL/min/1.73m^2^ |  |  |  |  |  |
| Mean (SD) | 43.4 (15.7) | 39.1 (16.0) | **<0.001** | 0.27 |  |
| Median (interquartile range) | 42.9 (32.0-53.6) | 37.7 (27.4-48.0) | **<0.001** |  |  |
| Range | 6.9-115.2 | 5.8-116.9 |  |  |  |
| Missing, n (%) | 0 (0.0) | 0 (0.0) |  |  |  |
| Urine protein-to-creatinine ratio, g/g |  |  |  |  |  |
| Mean (SD) | 0.8 (1.9) | 1.4 (3.2) | **<0.001** | 0.22 |  |
| Median (interquartile range) | 0.1 (0.1-0.7) | 0.2 (0.1-1.3) | **<0.001** |  |  |
| Range | 0.0-24.3 | 0.0-37.8 |  |  |  |
| Missing, n (%) | 44 (1.6) | 27 (3.2) |  |  |  |
| High-density lipoprotein cholesterol (mg/dL) |  |  |  |  |  |
| Mean (SD) | 48.7 (15.7) | 47.1 (15.8) | **<0.01** | 0.1 |  |
| Median (interquartile range) | 46.0 (38.0-56.0) | 44.0 (36.0-55.0) | **<0.01** |  |  |
| Range | 7.0-158.0 | 9.0-174.0 |  |  |  |
| Missing, n (%) | 2 (0.1) | 2 (0.2) |  |  |  |
| Low-density lipoprotein cholesterol (mg/dL) |  |  |  |  |  |
| Mean (SD) | 100.5 (34.2) | 98.2 (37.0) | 0.11 | 0.07 |  |
| Median (interquartile range) | 96.0 (77.0-120.0) | 95.5 (73.0-116.0) | **<0.05** |  |  |
| Range | 17.0-285.0 | 21.0-313.0 |  |  |  |
| Missing, n (%) | 3 (0.1) | 2 (0.2) |  |  |  |
| Hemoglobin (g/dL) |  |  |  |  |  |
| Mean (SD) | 12.9 (1.8) | 12.5 (1.8) | **<0.001** | 0.19 |  |
| Median (interquartile range) | 12.8 (11.6-14.1) | 12.5 (11.2-13.7) | **<0.001** |  |  |
| Range | 6.9-19.5 | 7.0-18.9 |  |  |  |
| Missing, n (%) | 1 (0.0) | 1 (0.1) |  |  |  |
| Glycosylated hemoglobin (%) |  |  |  |  |  |
| Mean (SD) | 6.5 (1.4) | 6.7 (1.6) | **<0.001** | 0.16 |  |
| Median (interquartile range) | 6.0 (5.6-7.0) | 6.2 (5.6-7.3) | **<0.001** |  |  |
| Range | 3.8-14.4 | 3.5-13.3 |  |  |  |
| Missing, n (%) | 33 (1.2) | 19 (2.3) |  |  |  |
| Brain natriuretic peptide (pg/mL) |  |  |  |  |  |
| Mean (SD) | 75.9 (163.7) | 148.0 (375.0) | **<0.001** | 0.25 |  |
| Median (interquartile range) | 37.0 (15.7-83.3) | 53.8 (21.9-132.7) | **<0.001** |  |  |
| Range | 1.5-4773.5 | 5.0-6972.9 |  |  |  |
| Missing, n (%) | 36 (1.3) | 15 (1.8) |  |  |  |
| N-terminal pro b-type natriuretic peptide (pg/mL) |  |  |  |  |  |
| Mean (SD) | 364.4 (1032.8) | 913.6 (2760.0) | **<0.001** | 0.26 |  |
| Median (interquartile range) | 129.2 (57.4-324.4) | 212.6 (83.5-680.1) | **<0.001** |  |  |
| Range | 2.5-24910.0 | 2.5-35000.0 |  |  |  |
| Missing, n (%) | 53 (2.0) | 22 (2.6) |  |  |  |
| **Medications, n (%)** |  |  |  |  |  |
| ACE Inhibitors | 1302 (48.7) | 371 (44.8) | **<0.05** | 0.03 |  |
| Alpha 2 Agonists | 221 (8.3) | 87 (10.5) | **<0.05** | 0.03 |  |
| Angiotensin Receptor Blockers | 720 (26.9) | 235 (28.3) | 0.43 | 0.01 |  |
| Alpha Blockers | 396 (14.8) | 152 (18.3) | **<0.05** | 0.04 |  |
| Beta Blockers | 1287 (48.1) | 475 (57.3) | **<0.001** | 0.08 |  |
| Calcium Channel Blockers | 1067 (39.9) | 370 (44.6) | **<0.05** | 0.04 |  |
| Antiplatelet Drugs | 1304 (48.8) | 426 (51.4) | 0.19 | 0.02 |  |
| Digoxin | 77 (2.9) | 42 (5.1) | **<0.01** | 0.05 |  |
| Loop Diuretics | 956 (35.8) | 395 (47.6) | **<0.001** | 0.1 |  |
| Thiazide Diuretics | 720 (26.9) | 208 (25.1) | 0.29 | 0.02 |  |
| Potassium Sparing Diuretics | 225 (8.4) | 62 (7.5) | 0.39 | 0.01 |  |
| Coronary Vasodilators | 192 (7.2) | 83 (10.0) | **<0.01** | 0.04 |  |
| Statins | 1580 (59.1) | 489 (59.0) | 0.95 | 0 |  |
| Other Non-Statin Lipid-Lowering Drugs | 434 (16.2) | 122 (14.7) | 0.3 | 0.02 |  |
| NSAIDs | 1416 (53.0) | 452 (54.5) | 0.43 | 0.01 |  |
| Aspirin | 1216 (45.5) | 397 (47.9) | 0.23 | 0.02 |  |
| Anti-Diabetes Therapy | 720 (26.9) | 241 (29.1) | 0.23 | 0.02 |  |
| **Year 1 Echo Measurements** |  |  |  |  |  |
| Aortic Valve Continuous Wave Peak Velocity (cm/sec) |  |  |  |  |  |
| Mean (SD) | 121.8 (31.7) | 125.8 (35.8) | **<0.01** | 0.12 |  |
| Median (interquartile range) | 117.0 (102.0-134.0) | 121.0 (103.0-138.0) | **<0.05** |  |  |
| Range | 54.0-390.0 | 55.0-356.0 |  |  |  |
| Missing, n (%) | 131 (4.9) | 47 (5.6) |  |  |  |
| Degree of Aortic Regurgitation (categorical) |  |  | 0.32 | 0.04 |  |
| None | 2098 (78.5) | 644 (77.4) |  |  |  |
| Mild | 452 (16.9) | 147 (17.7) |  |  |  |
| Moderate | 56 (2.1) | 22 (2.6) |  |  |  |
| Severe | 1 (0.0) | 2 (0.2) |  |  |  |
| Missing | 66 (2.5) | 17 (2.0) |  |  |  |
| Degree of Mitral Regurgitation (categorical) |  |  | **<0.01** | 0.07 |  |
| None | 652 (24.4) | 223 (26.8) |  |  |  |
| Mild | 1782 (66.7) | 506 (60.8) |  |  |  |
| Moderate | 132 (4.9) | 63 (7.6) |  |  |  |
| Severe | 17 (0.6) | 10 (1.2) |  |  |  |
| Missing | 90 (3.4) | 30 (3.6) |  |  |  |
| Left ventricular end-diastolic volume indexed to BSA |  |  |  |  |  |
| Mean (SD) | 67.8 (17.8) | 71.1 (21.2) | **<0.001** | 0.17 |  |
| Median (interquartile range) | 65.1 (56.3-76.6) | 67.8 (57.1-80.9) | **<0.01** |  |  |
| Range | 27.8-272.9 | 34.3-186.5 |  |  |  |
| Missing, n (%) | 77 (2.9) | 23 (2.8) |  |  |  |
| Diastolic Relaxation |  |  | **<0.05** | 0.06 |  |
| Normal | 700 (26.2) | 216 (26.0) |  |  |  |
| Mildly Abnormal | 1485 (55.6) | 479 (57.6) |  |  |  |
| Moderately Abnormal | 211 (7.9) | 50 (6.0) |  |  |  |
| Severely Abnormal | 25 (0.9) | 17 (2.0) |  |  |  |
| Missing | 252 (9.4) | 70 (8.4) |  |  |  |
| Left Ventricular Ejection Fraction, %, Categorical |  |  | **<0.001** | 0.09 |  |
| >=50 | 2094 (78.3) | 604 (72.6) |  |  |  |
| 40-49 | 352 (13.2) | 119 (14.3) |  |  |  |
| <40 | 146 (5.5) | 86 (10.3) |  |  |  |
| Missing | 81 (3.0) | 23 (2.8) |  |  |  |
| Left Ventricular Ejection Fraction, % |  |  |  |  |  |
| Mean (SD) | 54.5 (8.1) | 53.0 (9.8) | **<0.001** | 0.16 |  |
| Median (interquartile range) | 55.3 (51.4-59.0) | 54.8 (49.9-58.6) | **<0.01** |  |  |
| Range | 15.5-76.8 | 11.0-77.7 |  |  |  |
| Missing, n (%) | 81 (3.0) | 23 (2.8) |  |  |  |
| Interventricular Septal Thickness in diastole (cm) |  |  |  |  |  |
| Mean (SD) | 1.4 (0.3) | 1.4 (0.4) | **<0.001** | 0.22 |  |
| Median (interquartile range) | 1.3 (1.1-1.6) | 1.4 (1.2-1.7) | **<0.001** |  |  |
| Range | 0.6-2.6 | 0.4-2.8 |  |  |  |
| Missing, n (%) | 239 (8.9) | 70 (8.4) |  |  |  |
| Left Atrial Four Chamber Area (cm^2^) |  |  |  |  |  |
| Mean (SD) | 23.1 (5.5) | 24.2 (6.2) | **<0.001** | 0.19 |  |
| Median (interquartile range) | 22.5 (19.1-26.4) | 23.5 (19.8-28.1) | **<0.001** |  |  |
| Range | 8.2-46.4 | 9.6-55.4 |  |  |  |
| Missing, n (%) | 42 (1.6) | 16 (1.9) |  |  |  |
| Left Ventricular Geometry |  |  | **<0.001** | 0.1 |  |
| Normal | 474 (17.7) | 98 (11.8) |  |  |  |
| Concentric Remodeling | 645 (24.1) | 164 (19.7) |  |  |  |
| Eccentric Hypertrophy | 321 (12.0) | 115 (13.8) |  |  |  |
| Concentric Hypertrophy | 781 (29.2) | 307 (36.9) |  |  |  |
| Missing | 452 (16.9) | 148 (17.8) |  |  |  |
| Left Ventricular Internal Diameter-Diastole (cm) |  |  |  |  |  |
| Mean (SD) | 4.6 (0.7) | 4.6 (0.7) | 0.29 | 0.04 |  |
| Median (interquartile range) | 4.5 (4.1-5.0) | 4.6 (4.1-5.0) | 0.44 |  |  |
| Range | 2.6-8.2 | 2.5-7.6 |  |  |  |
| Missing, n (%) | 213 (8.0) | 63 (7.6) |  |  |  |
| Left Ventricular Internal Diameter-Systole (cm) |  |  |  |  |  |
| Mean (SD) | 3.2 (0.7) | 3.3 (0.8) | **<0.05** | 0.09 |  |
| Median (interquartile range) | 3.1 (2.7-3.6) | 3.1 (2.7-3.6) | 0.21 |  |  |
| Range | 1.2-7.4 | 1.0-7.0 |  |  |  |
| Missing, n (%) | 263 (9.8) | 78 (9.4) |  |  |  |
| Left Ventricular Mass indexed to BSA (g/m^2^) |  |  |  |  |  |
| Mean (SD) | 102.3 (24.4) | 109.4 (27.8) | **<0.001** | 0.27 |  |
| Median (interquartile range) | 98.8 (85.3-115.2) | 105.6 (89.3-124.8) | **<0.001** |  |  |
| Range | 39.4-212.2 | 46.2-229.9 |  |  |  |
| Missing, n (%) | 358 (13.4) | 117 (14.1) |  |  |  |
| Posterior wall thickness in diastole (cm) |  |  |  |  |  |
| Mean (SD) | 1.1 (0.2) | 1.2 (0.2) | **<0.001** | 0.15 |  |
| Median (interquartile range) | 1.1 (1.0-1.3) | 1.2 (1.0-1.3) | **<0.001** |  |  |
| Range | 0.4-2.2 | 0.6-2.2 |  |  |  |
| Missing, n (%) | 237 (8.9) | 72 (8.7) |  |  |  |
| Relative wall thickness |  |  |  |  |  |
| Mean (SD) | 0.5 (0.1) | 0.5 (0.1) | **<0.05** | 0.1 |  |
| Median (interquartile range) | 0.5 (0.4-0.6) | 0.5 (0.4-0.6) | **<0.01** |  |  |
| Range | 0.2-1.2 | 0.2-1.1 |  |  |  |
| Missing, n (%) | 237 (8.9) | 72 (8.7) |  |  |  |
| Relative wall thickness in diastole |  |  |  |  |  |
| Mean (SD) | 0.5 (0.1) | 0.5 (0.1) | **<0.05** | 0.1 |  |
| Median (interquartile range) | 0.5 (0.4-0.6) | 0.5 (0.4-0.6) | **<0.01** |  |  |
| Range | 0.2-1.2 | 0.2-1.1 |  |  |  |
| Missing, n (%) | 237 (8.9) | 72 (8.7) |  |  |  |
| Left ventricular end-systolic volume (ml) indexed to BSA |  |  |  |  |  |
| Mean (SD) | 31.5 (13.7) | 34.6 (17.7) | **<0.001** | 0.19 |  |
| Median (interquartile range) | 28.9 (23.9-35.6) | 30.1 (24.2-38.2) | **<0.001** |  |  |
| Range | 9.3-221.2 | 10.5-159.9 |  |  |  |
| Missing, n (%) | 81 (3.0) | 23 (2.8) |  |  |  |
